# Physicians’ Perceptions of Chatbots in Health Care: Cross-Sectional Web-Based Survey

**DOI:** 10.2196/12887

**Published:** 2019-04-05

**Authors:** Adam Palanica, Peter Flaschner, Anirudh Thommandram, Michael Li, Yan Fossat

**Affiliations:** 1 Labs Department Klick Health Klick Inc Toronto, ON Canada; 2 Marketing Department Klick Health Klick Inc Toronto, ON Canada

**Keywords:** physician satisfaction, health care, telemedicine, mobile health, health surveys

## Abstract

**Background:**

Many potential benefits for the uses of chatbots within the context of health care have been theorized, such as improved patient education and treatment compliance. However, little is known about the perspectives of practicing medical physicians on the use of chatbots in health care, even though these individuals are the traditional benchmark of proper patient care.

**Objective:**

This study aimed to investigate the perceptions of physicians regarding the use of health care chatbots, including their benefits, challenges, and risks to patients.

**Methods:**

A total of 100 practicing physicians across the United States completed a Web-based, self-report survey to examine their opinions of chatbot technology in health care. Descriptive statistics and frequencies were used to examine the characteristics of participants.

**Results:**

A wide variety of positive and negative perspectives were reported on the use of health care chatbots, including the importance to patients for managing their own health and the benefits on physical, psychological, and behavioral health outcomes. More consistent agreement occurred with regard to administrative benefits associated with chatbots; many physicians believed that chatbots would be most beneficial for scheduling doctor appointments (78%, 78/100), locating health clinics (76%, 76/100), or providing medication information (71%, 71/100). Conversely, many physicians believed that chatbots cannot effectively care for all of the patients’ needs (76%, 76/100), cannot display human emotion (72%, 72/100), and cannot provide detailed diagnosis and treatment because of not knowing all of the personal factors associated with the patient (71%, 71/100). Many physicians also stated that health care chatbots could be a risk to patients if they self-diagnose too often (714%, 74/100) and do not accurately understand the diagnoses (74%, 74/100).

**Conclusions:**

Physicians believed in both costs and benefits associated with chatbots, depending on the logistics and specific roles of the technology. Chatbots may have a beneficial role to play in health care to support, motivate, and coach patients as well as for streamlining organizational tasks; in essence, chatbots could become a surrogate for nonmedical caregivers. However, concerns remain on the inability of chatbots to comprehend the emotional state of humans as well as in areas where expert medical knowledge and intelligence is required.

## Introduction

### Background

Chatbots, also known as conversational agents, interactive agents, virtual agents, virtual humans, or virtual assistants, are artificial intelligence programs designed to simulate human conversation via text or speech. Many positive viewpoints have been made on the potential uses of health care chatbots within the marketing and business world [1-10]; however, little scientific research has examined their effectiveness in real-world patient scenarios, that is, to improve health outcomes [11,12]. Chatbots are commonly used in marketing applications such as to guide consumers through electronic commerce websites, answer questions related to products and services, help troubleshoot problems with internet service, act as a personal concierge, or provide consumer advice. In the context of health care, chatbots or *healthbots* are intended to provide personalized health and therapy information to patients, provide relevant products and services to patients, as well as suggest diagnoses and recommend treatments based on patient symptoms.

Chatbots in health care may have the potential to provide patients with access to immediate medical information, recommend diagnoses at the first sign of illness, or connect patients with suitable health care providers (HCPs) across their community [13,14]. Theoretically, in some instances, chatbots may be better suited to help patient needs than a human physician because they have no biological gender, age, or race and elicit no bias toward patient demographics. Chatbots do not get tired, fatigued, or sick, and they do not need to sleep; they are cost-effective to operate and can run 24 hours a day, which is especially useful for patients who may have medical concerns outside of their doctor’s operating hours. Chatbots can also communicate in multiple different languages to better suit the needs of individual patients.

Early research has demonstrated the benefits of using health care chatbots, such as helping with diagnostic decision support [15,16], promoting and increasing physical activity [17], and cognitive behavioral therapy for psychiatric and somatic disorders [18-24], which provide effective, acceptable, and practical health care with accuracy comparable with that of human physicians. Patients may also feel that chatbots are *safer* interaction partners than human physicians and are willing to disclose more medical information and report more symptoms to chatbots [25,26]. However, despite the demonstrated efficacy and cost-effectiveness of health care chatbots, the technology is usually associated with poor adoption by physicians and poor adherence by patients [27]. This may be because of the perceived lack of quality or accountability that is characterized by computerized chatbots as opposed to traditional face-to-face interactions with human physicians.

### Objectives

Although there are some instances showing the effectiveness of health care–related chatbots for certain outcomes, it is still not entirely clear whether this technology is better overall in improving all the various clinical health outcomes of patients and why it is not more highly adopted compared with traditional methods of care, that is, information coming from a human physician. Although chatbot technology for health care is continually advancing, little is known about the perspectives of practicing medical physicians on the use of chatbots in health care. It would thus seem beneficial to have medical expert opinions on the use of this technology that is intended to supplement or even replace specific roles of HCPs. The purpose of this study was to examine the perspectives of practicing medical physicians on the use of health care chatbots for patients. As human physicians have been the traditional benchmark for treating patients for hundreds of years, a crucial objective of investigating the use of chatbots for delivering health care should be to understand the perspective of medical experts who actually practice health care in their daily occupations. As physicians are the primary point of care for patients, their approval is an important gate to the dissemination of chatbots into medical practice. The findings of this research will help to either justify or attenuate enthusiasm for health care chatbot applications as well as direct future work to better align with the needs of HCPs.

## Methods

### Participants

A total of 100 participants completed the survey (28 females, 69 males, and 3 preferred not to say; age range=28-73 years; mean age 44.9, SD 12.0). Participants were general practitioners (GPs) with a Doctor of Medicine (MD) degree (years of practice range=2-36; mean years of practice 14.7, SD 9.0). Participants were located across 32 states of the United States, with participants in each of the 4 main interstate regions of Northeast (27/100, 27%; 5 states), Midwest (21/200, 21%; 8 states), South (29/100, 29%; 13 states), and West (23/100, 23%; 6 states).

### Recruitment

Participants who took part in the survey were sampled from a large database of physicians who have previously agreed to take part in market research. The survey was administered by Sermo [28], a private social media network for licensed physicians, who randomly selected registered physicians within their panel across the United States. The Sermo research network comprises over 400,000 registered physicians in the United States, representing roughly 40% of the US physician population [19]. As this study was the first of its kind and exploratory in nature to study the subjective opinions of physicians, no explicit statistical hypotheses were being evaluated. The sample size of 100 was arbitrarily chosen to gather a preliminary viewpoint of physicians’ perspectives of chatbots in health care and would yield approximately a 9.8% margin of error with a 95% CI of the entire US physician population.

Invitees were sent an email inviting them to complete the survey, accessible via an embedded Web link. The only inclusion criteria included being a GP with an MD degree within the United States; no restrictions on age, gender, or previous use of chatbots were implemented. Exclusion criteria included any nonphysician or specialist physician, which may have had a bias toward the use of health care chatbots for patients; in contrast, GPs are typically the primary point of care for a broad range of family medicine–related health issues, so these participants were targeted for the research. All participants gave informed consent to complete the survey, and the study received full ethics clearance from Advarra Institutional Review Board Services, an independent ethics committee.

### Survey and Procedure

Survey questions were designed in consultation with medical scientists, Web developers, data scientists, and technology specialists with expertise in digital medicine. The survey was organized into 5 main sections, including (1) usage of health care chatbots, (2) perceived benefits of health care chatbots to patients, (3) perceived challenges of health care chatbot usage, (4) perceived risks of health care chatbots, and (5) physicians’ perceptions of health care chatbots in the role of a physician.

Participants were asked to take part in a research study involving a Web-based self-report survey that examines physician perceptions of the benefits, challenges, and risks of using chatbots in health care. Participants were asked to provide demographic information and report their opinions on the use of chatbot technology for treating patients in health care. At the beginning of the survey, participants were given an explicit definition of *chatbot*, to give a baseline description for the context of the questions in the survey. Participants were given the following definition:

Chatbots, also known as conversational agents, interactive agents, virtual agents, virtual humans, or virtual assistants, are computer software applications that run automated tasks or scripts designed to simulate human conversation. Chatbots are artificial intelligence (AI) programs that can generate and retrieve information for the interaction with human users via text or computer voice generation.

Participants were asked to answer all the survey questions for chatbots in the context of health care, referring to the use of chatbots for health-related issues.

### Data Analysis

Data were analyzed using descriptive statistics and frequencies to examine the characteristics of participant responses to survey items on health care chatbots. Preliminary analyses revealed no major differences across factors of age, gender, or years of practice. Thus, the entire sample was reported holistically.

## Results

### Usage of Health Care Chatbots

A total of 30% (30/100) of participants indicated that they had direct personal experience with the use of chatbots for health-related issues. Physicians were also given a list of currently available health care chatbots, to examine their familiarity with some of the interfaces that could be potentially accessed by patients. Table 1 shows physicians’ use of these health care chatbots, which are intended to provide personalized health and therapy information, provide relevant products and services to patients, as well as suggest diagnoses and recommend treatments based on patients’ symptoms. The findings indicated that most of the currently available chatbots were not generally used or heard of by physicians.

Of the 30 participants who have used health care chatbots previously, 4 (13%) were very satisfied, 10 (33%) were somewhat satisfied, 8 (27%) were neither satisfied nor dissatisfied, and 8 (27%) were somewhat dissatisfied with their application. Of all the physicians in the survey, 18% (18/100) stated that their patients use health care chatbots (24%, 24/100, stated that patients did not use them), but the majority (58%, 58/100) were unsure or did not know whether their patients use them.

In total, 42% (42/100) of physicians believed that chatbots are either very important (9%, 9/100) or somewhat important (33%, 33/100) in health care, whereas 26% (26/100) believed that they are somewhat unimportant (18%, 18/100, 18%) or very unimportant (8%, 8/100); 32% (32/100) of physicians believed that they are neither important nor unimportant. Similarly, 44% (44/100) of physicians stated that they would be very likely (9/100, 9%) or somewhat likely (35%, 35/100) to prescribe the use of health care chatbots to their patients within the next 5 years; 34% (34/100) of physicians stated that they would be somewhat unlikely (22/100, 22%) or very unlikely (12/100, 12%) to do so. A total of 40% (40/100) of physicians also indicated that they would be very likely (11/100, 11%) or somewhat likely (29%, 29/100) to recommend the prescription of health care chatbots to their HCP colleagues, whereas 37% (37/100) indicated that they would be somewhat unlikely (25%, 25/100) or very unlikely (12%, 12/100) to do the same.

### Perceived Benefits of Health Care Chatbots to Patients

Participants were asked to what extent they thought health care chatbots would benefit patients in specific areas of health management (Table 2). An average of 42% (42/100) agreed to some extent in the benefits associated with health care chatbots, whereas an average of 25% (25/100) disagreed to some extent in these same potential benefits. More than half of physicians agreed that health care chatbots could help patients better manage their own health (54/100, 54%), improve access and timeliness to care (53%, 53/100), or reduce travel time to their HCP (52%, 52/100); almost half of physicians believed that health care chatbots could prevent unnecessary visits to HCPs (49/100, 49%) or that patients may disclose more information to chatbots compared with HCPs (41%, 41/100).

In terms of specific health-related outcomes of chatbot use for patients, an average of 45% (45/100) of physicians believed in some type of physical, psychological, or behavioral health benefit to patients (Table 3). More than half of physicians believed that health care chatbots could improve nutrition or diet (65%, 65/100), enhance medication or treatment adherence (60%, 60/100), increase activity or exercise (55%, 55/100), or reduce stress (51%, 51/100).

**Table 1:** Participants’ use of currently available health care chatbots.

Currently available health care chatbots^a^	Used it, %	Heard of it but never used it, %	Neither heard of it nor used it, %
Your.MD	14	21	65
HealthTap	11	17	72
Cancer Chatbot	8	13	79
VitaminBot	7	13	80
Babylon Health	5	14	81
Safedrugbot	5	10	85
Ada Health	3	12	85
Bots4Health	3	14	83
SimSensei	3	12	85
Sensely	2	12	86
Infermedica	2	16	82
Izzy	2	11	87
Luma Health	2	14	84
Ginger.io	2	12	86
Buoy Health	1	14	85
Florence	1	9	90
HelloJoy	1	11	88
Woebot	0	13	87
GYANT	0	9	91

^a^For simplicity, as there were exactly 100 participants in the study, only percentages have been reported, unless otherwise stated.

**Table 2:** Perceived benefits of health care chatbots to patients.

Perceived benefits of health care chatbots^a^	Strongly agree, %	Somewhat agree, %	Neither agree nor disagree, %	Somewhat disagree, %	Strongly disagree, %
Help patients better manage their own health	7	47	26	17	3
Improve quality of patient care	8	27	36	21	8
Help provide more personalized treatment	7	21	36	27	9
Reduce travel time to health care provider	20	32	30	12	6
Prevent unnecessary visits to health care providers	11	38	28	19	4
Patients may disclose more information to chatbots compared with health care providers	12	29	36	15	8
Increase patient privacy	8	19	40	20	13
Improve access and timeliness to care	15	38	32	12	3
Average across variables	11	31	33	18	7

^a^For simplicity, as there were exactly 100 participants in the study, only percentages have been reported, unless otherwise stated.

**Table 3:** Perceived health care outcome benefits of using chatbots for patients.

Perceived health care outcome benefits of using chatbots^a^	Yes, %	No, %	Do not know or not sure, %
Activity or exercise increase	55	25	20
Alcohol consumption reduction	31	36	33
Blood glucose reduction	49	26	25
Blood pressure reduction	34	32	34
Cholesterol reduction	32	37	31
Cognitive behavioral therapy	41	30	29
Depression reduction	33	37	30
Medication/treatment adherence	60	17	23
Nutrition/diet improvement	65	19	16
Sleep quality or quantity improvement	34	35	31
Smoking reduction or cessation	47	24	29
Stress reduction or management	51	23	26
Psychological well-being increase	48	28	24
Weight loss or decrease in body mass index	45	30	25
Average across variables	45	29	27

^a^For simplicity, as there were exactly 100 participants in the study, only percentages have been reported, unless otherwise stated.

**Table 4:** Perceived logistical benefits of using chatbots for patients.

Perceived logistical benefits of using chatbots^a^	Yes, %	No, %	Do not know or not sure, %
Locating health clinics or health care providers in a specific area	76	11	13
Scheduling doctor appointments	78	13	9
Monitoring patient calls to the reception desk of health clinics	49	22	29
Processing medical invoices or bill payments	48	28	24
Assessing emergency triage in hospitals	30	48	22
Reminders for medication/treatment compliance	76	11	13
Renewing medication prescriptions	56	25	19
Gathering health insurance information	65	16	19
Answering medication frequently asked questions	70	15	15
Providing medication side effects and drug interactions	68	15	17
Providing medication use or misuse instructions	71	12	17
Average across variables	62	20	18

^a^For simplicity, as there were exactly 100 participants in the study, only percentages have been reported, unless otherwise stated.

Regarding logistical benefits of using health care chatbots for patients, the majority of physicians (62%, 62/100) believed in advantages for organization, planning, and management of administrative characteristics associated with health care (Table 4). Most notably, many physicians believed that chatbots would be most beneficial for scheduling doctor appointments (78%, 78/100), locating health clinics or HCPs in a specific area (76%, 76/100), administering reminders for medication or treatment compliance (76%, 76/100), providing medication use or misuse instructions (71%, 71/100), or answering medication frequently asked questions (70%, 70/100). In contrast, almost half of physicians believed that chatbots could *not* assess emergency triage in hospitals (48%, 48/100).

### Perceived Challenges Associated With Health Care Chatbots for Patients

Over half of the physicians (53%, 53/100) agreed to some extent in various challenges associated with the use of health care chatbots for patients, whereas an average of only 14% (14/100) disagreed with these traits (Table 5). Most notably, 76% (76/100) of physicians believed that chatbots cannot effectively care to the full extent of the patients’ needs, 72% (72/100) believed that chatbots cannot understand or display human emotion, and 58% (58/100) believed that chatbots lack the intelligence or knowledge to accurately assess patients.

**Table 5:** Perceived challenges associated with using health care chatbots for patients.

Perceived challenges associated with using health care chatbots^a^	Strongly agree, %	Somewhat agree, %	Neither agree nor disagree, %	Somewhat disagree, %	Strongly disagree, %
Patient data privacy and confidentiality	17	31	39	11	2
Chatbots cannot understand or display human emotion	36	36	23	4	1
Chatbots lack the intelligence or knowledge to accurately assess patients	19	39	27	11	4
Chatbots offer poor health-related advice	12	28	47	13	0
Chatbots cannot effectively care to the full extent of the patients’ needs	43	33	17	7	0
Chatbots take too much time to use	6	24	48	20	2
Most of my patients do not have access to the necessary technology for chatbots services	15	35	26	18	6
Average across variables	21	32	32	12	2

^a^For simplicity, as there were exactly 100 participants in the study, only percentages have been reported, unless otherwise stated.

**Table 6:** Perceived risks associated with using health care chatbots for patients.

Perceived risks associated with using health care chatbots	Strongly agree, %	Somewhat agree, %	Neither agree nor disagree, %	Somewhat disagree, %	Strongly disagree, %
Patients may abuse the use of chatbots and self-diagnose too often	21	53	20	5	1
Patients will receive lesser quality assessments	24	38	28	8	2
Patients may not accurately understand the diagnoses	26	48	22	2	2
Chatbots cannot provide detailed clarification on patient assessment	35	36	19	8	2
Patients may not feel adequately connected to their health care providers	34	36	22	8	0
Chatbots may indirectly harm patients by not knowing all of the personal factors associated with the patient	28	41	24	6	1
Average across variables	28	42	23	6	1

^a^For simplicity, as there were exactly 100 participants in the study, only percentages have been reported, unless otherwise stated.

### Perceived Risks Associated With Health Care Chatbots for Patients

The great majority of physicians (70%, 70/100) expressed their concern of risks associated with health care chatbots for patients, whereas only 7% (7/100) disagreed with these potential risks (Table 6). Over 60% (60/100) of physicians agreed to every type of risk presented to them, including the perception that patients may abuse the use of chatbots and self-diagnose too often (74%, 74/100), patients may not accurately understand the diagnoses (74%, 74/100), and that chatbots cannot provide detailed clarification on patient assessment (71%, 71/100). Physicians also felt that patients may not feel adequately connected to their HCPs (70%, 70/100) or that chatbots may indirectly harm patients by not knowing all of the personal factors associated with the patient (69%, 69/100).

### Physicians’ Perceptions of Health Care Chatbots in the Role of a Physician

Physicians were asked how much they believed that health care chatbots would either help them or impede their work in their daily occupational role on a sliding scale from 0%=*impede my work* to 100%=*help me.* Physicians responded entirely across the board, which averaged at an approximate neutral point in the middle of the scale (observed range=0-100%, mean 47.4%, median 50%, SD 25.6%).

Finally, physicians were asked how likely it would be, in the future, for health care chatbots to play a more significant role in patients’ health than their HCP. A total of 49% (49/100) expressed that this would be very likely (15%, 15/100) or somewhat likely (34%, 34/100) to happen, whereas 25% (25/100) expressed that this would be somewhat unlikely (15%, 15/100) or very unlikely (10%, 10/100) to happen.

## Discussion

### Principal Findings

A total of 100 practicing GPs participated in an online research survey that examined their perceived benefits, challenges, and risks of using chatbots in health care. Overall, the findings demonstrated that physicians have a wide variety of perspectives on the use of health care chatbots for patients, with few major skews to one side or the other regarding agreement levels to a variety of characteristics. Almost half of the physicians perceived health care chatbots to be important for patients, especially for helping patients better manage their own health. Almost half of the physicians also stated that they would be likely to prescribe the use of the technology to patients and recommend it to their colleagues. About half of the physicians also agreed that chatbots would benefit the physical, psychological, and behavioral health outcomes of patients, such as diet improvement, medication adherence, exercise frequency, or stress reduction. The other half of physicians was roughly equally divided between being an opponent or having a neutral opinion to the perceived importance and benefits of health care chatbots. In addition, patient privacy did not emerge as a polarizing issue.

With regard to the use of health care chatbots within the occupational role of an HCP, physicians believed that the technology would almost equally help them as well as impede their overall workplace duties. Approximately half of the physicians also believed that health care chatbots would eventually play a more significant role in patients’ health than their HCP. For the most part, these results indicated an almost equal number of supporters for health care chatbots, with the rest being those who are either indifferent or opponents to the technology.

More consistent agreement on the use of health care chatbots was apparent with reference to their potential logistical benefits as well as their challenges and potential risks to patients. For example, the great majority of physicians believed in administrative benefits associated with chatbots, especially for scheduling doctor appointments, locating health clinics or HCPs, administering reminders for medication compliance, providing treatment instructions, and answering commonly asked medication questions. In contrast, the majority of physicians believed that chatbots cannot effectively care for all of the patients’ needs, cannot understand or display human emotion, lack the intelligence to accurately assess patients, cannot provide detailed clarification on patient assessment, cannot assess emergency health situations, or may indirectly harm patients by not knowing all of the personal factors associated with the patient. In addition, many physicians stated that health care chatbots will be associated with the risk that patients may self-diagnose too often, patients may not understand the diagnoses, or that patients may not feel adequately connected to their primary physician.

These findings highlight the perception that chatbot technology may be advantageous to use in less complicated roles, such as administrative and organizational tasks, but more challenges and risks may be associated with their use in complex roles that involve more personalized knowledge of the patient. This suggests that health care duties involving an *expert human touch* and those that need a high degree of accuracy are perceived to be a poor choice for chatbot use compared with receiving overall treatment from an actual physician.

The many perceived challenges and risks associated with health care chatbots would need to be addressed before the technology is widely endorsed by practicing physicians. These challenges may be because of concerns involving regulation and remuneration to the physicians, which supports other relevant research demonstrating that physicians are less likely to use telemedicine services if they are not adequately compensated for their time and effort [29-31]. Addressing the perceived barriers around health care chatbots would, therefore, require cooperation by health care institutions, policy makers, HCPs, and patients alike.

### Limitations

One limitation to this research is that it examined the subjective perceptions of GPs as opposed to specialist physicians, which may have had more experience and different opinions on the use of health care chatbots, depending on their roles in patient health. In addition, all physicians practiced within the United States, which may be associated with a different level of enthusiasm toward digital medicine technology compared with other countries and cultures. Some research and viewpoints on health care chatbots have been published from international researchers around the world [13-27]; however, to the best of our knowledge, this was the first study to examine physicians’ perspectives on the direct use of chatbots in their practice. Future research should examine how different samples of HCPs, in different environments, perceive health care chatbots for use with their patients.

### Conclusions

Physicians believe in both costs and benefits associated with chatbots, depending on the logistics and specific roles of the technology. The areas where physicians believed chatbots would be most helpful were in the improvement of nutrition, diet, and treatment compliance as well as logistical tasks such as scheduling appointments, locating clinics, and providing medication reminders. The major challenges perceived were an inability of chatbots to understand emotions and address the full extent of a patient’s needs. Physicians also agreed that there were significant risks associated with chatbots including inaccurate medical information. These findings suggest that physicians may be comfortable with using chatbots to automate simple logistical tasks but do not believe that chatbots are advanced enough to replace complex decision-making tasks requiring an expert medical opinion. This is not to say that health care chatbots have a particular stigma associated with them, but rather, this suggests that improvements are needed for future use to overcome the risks and challenges associated with the technology. Nevertheless, nearly half of the physicians believed that health care chatbots could replace a major role of human HCPs sometime in the future. However, chatbots can be best applied to help physicians rather than replace them. Chatbots are cost-effective to run and can automate repetitive administrative tasks, thus freeing time for physicians to provide higher quality, personalized, and empathetic care to their patients. This research lays the foundation for future investigations on the factors influencing physician adoption of chatbots. Providing physicians with evidence-based research on the advantages and disadvantages of this emerging technology will help inform them on the most appropriate use to complement their practice rather than impede their work.

## Abbreviations

GP: general practitioner
HCP: health care provider
MD: Doctor of Medicine
